# Laparoscopic adjustable banded roux-en-y gastric bypass as a *primary *procedure for the super-super-obese (body mass index > 60 kg/m^2^)

**DOI:** 10.1186/1471-2482-10-33

**Published:** 2010-11-14

**Authors:** Bruno Dillemans, Sebastiaan Van Cauwenberge, Sanjay Agrawal, Els Van Dessel, Jan-Paul Mulier

**Affiliations:** 1 Department of General Surgery, AZ Sint-Jan Hospital AV, Ruddershove 10, 8000 Brugge, Belgium; 2 Department of Bariatric Surgery, Homerton University Hospital, London, UK; 3 Department of Anaesthesia, AZ Sint-Jan Hospital AV, Ruddershove 10, 8000 Brugge, Belgium

## Abstract

**Background:**

Currently, there is no consensus opinion regarding the optimal procedure of choice in super-super-morbid obesity (Body mass index, BMI > 60 kg/m2). Roux-en-Y gastric bypass (RYGB) is associated with failure to achieve or maintain 50% excess weight loss (EWL) or BMI < 35 in approximately 15% of patients. Also, percent EWL is significantly less after 1-year in the super-super-obese group as compared with the less obese group and many patients are still technically considered to be obese (lowest post-surgical BMI > 35) following RYGB surgery in this group. The addition of adjustable gastric band (AGB) to RYGB has been reported as a revisional procedure but this combined bariatric procedure has not been explored as a primary operation.

**Methods:**

In a primary laparoscopic RYGB, an AGB is drawn around the gastric pouch through a small opening between the blood vessels on the lesser curve and the gastric pouch. The band is then fixed by suturing the gastric remnant to the gastric pouch both above and below the band to prevent slippage.

**Results:**

Between November 2009 and March 2010, 6 consecutive super-super-obese patients underwent a primary laparoscopic adjustable banded Roux-en-Y gastric bypass procedure at our institution. One male patient (21 years, BMI 70 kg/m²) developed a pneumonia postoperatively. No other postoperative complications were observed.

**Conclusion:**

To the best of our knowledge, this is the first series of patients that underwent a laparoscopic adjustable banded RYGB as a primary operation for the super-super obese in the indexed literature. With the combined procedure, a sequential action mechanism for weight loss is to be expected. The restrictive, malabsorptive and hormonal working mechanism of the RYGB will induce weight loss from the start reaching a stabilised plateau of weight after 12 - 18 months. At that time, filling of the band can be started resulting in further gastric pouch restriction and increased weight loss. Moreover, besides improving the results of total weight loss, a gradual filling of the band can as well prevent the RYGB patient from weight regain if restriction would fade away with time.

## Background

Currently, there is no consensus opinion regarding the surgical procedure of choice in super-super morbid obesity (Body mass index, BMI > 60 kg/m^2^). Following a debate during the 2005 annual meeting of the Society of American Gastrointestinal and Endoscopic Surgeons (SAGES), surgeons choose laparoscopic gastric bypass as the procedure of choice in a hypothetical super-super obese patient case scenario [1]. Unfortunately, there is very little published data examining and comparing the outcomes in super-super obese patients with any of the well known bariatric procedures.

Since its first description in open surgery in 1966 [2] and by laparoscopy in 1994 [3], Roux-en-Y gastric bypass (RYGB) has become one of the most popular surgical procedures for morbid obesity [4]. In spite of higher risk and technical challenges, laparoscopic RYGBP (LRYGBP) can be safely performed in the super-super-obese [5-7]. Unfortunately, RYGB is associated with failure to achieve or maintain 50% excess weight loss (EWL) or BMI < 35 in approximately 15% (5-40%) of patients [8-10]. Also, percent EWL is significantly less after 1-year in the super-super-obese group as compared with the less obese group and many patients are still technically considered to be obese (lowest post-surgical BMI > 35) following RYGB surgery in the super-super obese group [7,11].

In the case of poor weight loss or weight regain, surgical alternatives reported in the literature include conversion to a distal bypass [12,13] or BPD/DS [14]. Dapri et al has reported laparoscopic placement of a non-adjustable silicone ring in six patients for weight regain after RYGB with good results [15]. Recently, the addition of adjustable gastric band (AGB) around the gastric pouch either by laparoscopy [16,17] or by open access [18] has also been reported as a revisional procedure. However, revisional bariatric procedures are technically more complex and associated with increased postoperative complications [9,10,19].

Following encouraging results with adjustable gastric band as a revisional procedure [15-17] in patients with weight regain or poor initial weight loss after RYGB, we performed a combined procedure of laparoscopic adjustable gastric banding with Roux-en-Y gastric bypass as a primary operation for the super-super obese patient as described below.

## Methods

### Patients

Between November 2009 and March 2010, 6 consecutive super-super-obese patients (4 women and 2 men; mean age 40.5 years; range 21-48 years) underwent a primary laparoscopic adjustable banded Roux-en-Y gastric bypass procedure at our institution. Mean BMI at the time of operation was 70.7 kg/m^2 ^(range 69-73.9 kg/m^2^). Prior to surgery all patients followed a high protein diet for at least two weeks in order to achieve a reduction in visceral adipose tissue and liver volume [20]. All the patients were provided with information regarding the operation itself and possible complications. The patients consented with knowledge of routine postoperative complications and specific warning of band slippage, migration, and band or port infection. The first follow-up visit is scheduled for after 6 weeks. Thereafter, visits are planned 6, 12, and 24 months postoperatively.

### Surgical technique

**Figure 1:**
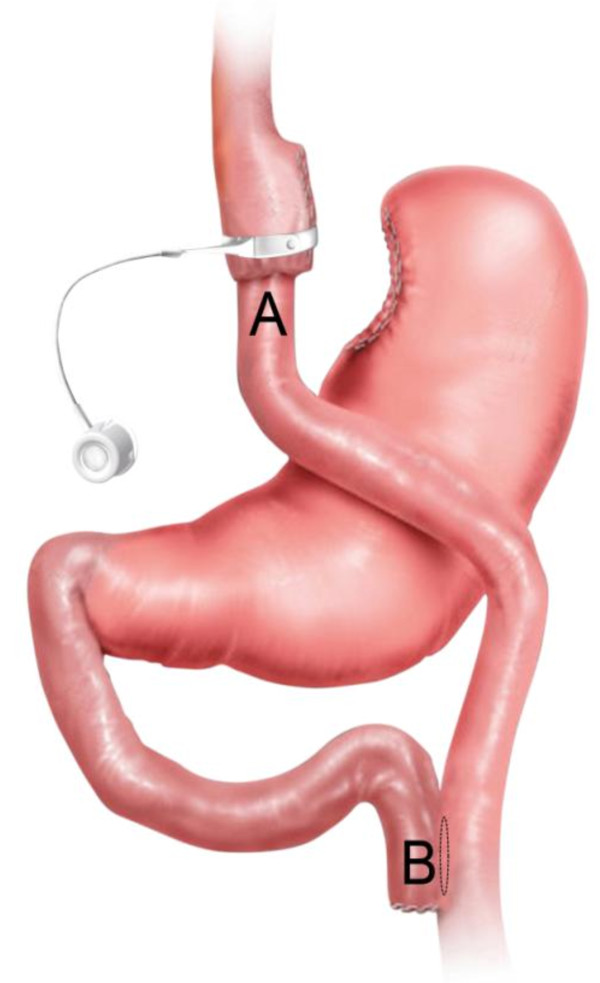
**Laparoscopic adjustable gastric banded Roux-en-Y gastric bypass**. Schematic representation of the Roux-en-Y gastric bypass construction with the adjustable band wrapped around the gastric pouch.

**Figure 2:**
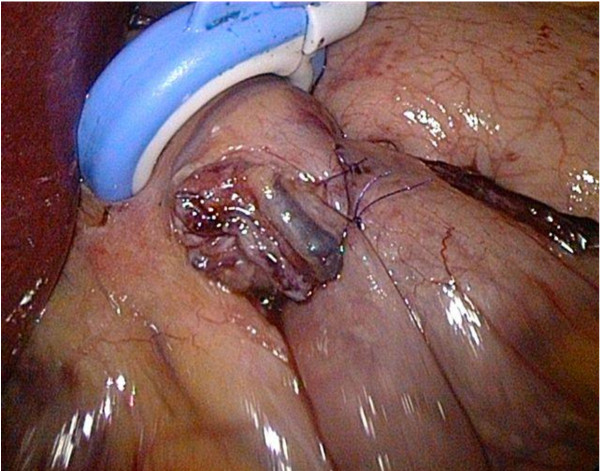
**Adjustable banded Roux-en-Y Gastric Bypass with a Heliogast® HAGB band around the gastric pouch**. Intra-operative view of the gastro-enterostomy with the band cranial of it.

One dose of cefazoline 1 g was given IV at induction of general anaesthesia. The patients were placed in the supine position, split-leg with reverse Trendelenberg position along with slight flexion of the hip to help increase surgical abdominal workspace [21]. The surgeon stood between the legs. A video monitor is positioned at the level of the patient's head. A 30° angle scope is used. Abdominal insufflation with carbon dioxide (CO2) is achieved using a Veress needle. Intra-abdominal pressures are maintained at 15 to 17 mmHg. A five-port technique was employed: a 10 mm port 10-15 cm below the xiphoid process, a 5 mm port high epigastric on the midline, a 12 mm port in the right upper quadrant and a 15 mm and 12 mm port in the left upper quadrant. The latter two ports are placed on the same line of the 10 mm port with the 15 mm port in the middle between the 10 mm and the 12 mm port. The former 12 mm port is placed somewhat higher above the same line (sub costal). Since we perform a standardized fully stapled laparoscopic RYGB procedure, the procedure started with the creation of the gastric pouch following the same principles as previously published by our group [22]. After creation of the gastric pouch, an atraumatic grasper was passed through a small opening between the blood vessels on the lesser curve and the gastric pouch 1-2 cm above the horizontal cut edge of the pouch. Following this step, an AGB was introduced via the 15 mm port, drawn around the pouch and locked into place (Figure 1). In the first four patients, a Heliogast® HAGE band (Helioscopie, France) has been placed. The last two patients received a newer type of band, the Heliogast® HAGB band (Helioscopie, France), which is easier to fit because of its lesser diameter and width (Figure 2).

The remaining part of the operation was completed as described [22]. Since all patients had a BMI > 50 kg/m^2^, the length of the alimentary limb was measured at 200 cm. To prevent slippage, the band was then fixed by suturing the gastric remnant to the gastric pouch both above and below the band with nonabsorbable sutures (2/0 Ethibond, Ethicon). The subcutaneous reservoir port was secured to the anterior rectus sheath in a midclavicular plane and the tubing of the band connected to the port. The band was not filled at the operation.

## Results

Mean operative time was 75 minutes (range 64-121 min); no conversions were performed. Mean hospital stay was 3.3 days. All patients were allowed fluids from the second postoperative day.

One male patient (21 years, BMI 70 kg/m^2^) developed a pneumonia postoperatively. He was given intravenous antibiotics along with intensive chest physiotherapy and was discharged home on the fifth postoperative day.

No other postoperative complications were observed. There was no mortality in the group. There were no band or port-related infections and all patients were doing extremely well at their first follow-up visit in the outpatient policlinic six weeks after the operation.

## Discussion

To the best of our knowledge, this is the first series of patients that underwent a laparoscopic adjustable gastric banded Roux-en-Y gastric bypass as a *primary *operation for the super-super obese in the indexed literature.

Prosthetic devices have been used in bariatric operations to control the outlet of the gastric pouch and thus induce restriction to help maintain weight loss. Initial devices commonly employed in vertical banded gastroplasties (VBG) included a fixed diameter silastic ring or even mesh prostheses. In 1991 the group of Capella was the first to describe the vertical banded gastroplasty-gastric bypass with a 5.5 cm supporting band around a small gastric pouch [23]. In that same year, Fobi et al published their results with the silastic ring vertical banded gastric bypass [24]. Although producing effective restriction, the non adjustability of these devices has led to problems that have been reported in several series. Salinas *et al *have reported the results on a series of 1588 patients following various modifications of the silastic ring vertical bypass. They report stricture rates up to 3.8% in one of their subgroups, and the necessity of ring removal in 5.7% of the total population [25]. In a study comparing two silastic ring sizes, Cramtpon and colleagues report eating problems in 28% of patients requiring removal in 14% of patients with a 5.5 cm diameter ring, and in 4% of patients with a 6 cm ring [26,27]. The latter finding provides support for the superiority of a variable diameter system allowing adjustment for patient tolerance [26]. Interestingly, Kyzer et al report good results following the use of AGB in a subgroup of 22 patients that previously had silastic ring gastroplasties [28]. There are similar small series following the conversion of a non adjustable band to an adjustable system [29]. The adjustability of the device should, at least theoretically, counteract the possible complications associated with the non-adjustable ring. In a long-term follow-up study comparing VBG to AGB, Miller et al demonstrated a statistically significant lower re-intervention and re-operation rate and an improved health status and quality of life for the AGB group [30].

There are reports of using an AGB with RYGB where the bands were placed below the gastro-jejunostomy to form the gastric pouch [31,32]. However, these operations had a high incidence of band erosions into the stomach and the small bowel. In an expert meeting on the adjustable banded gastric bypass at the 3^rd ^annual meeting of the Italian Collaborative Study Group for the Lap-Band (2003), it was concluded that the combination of gastric bypass with an AGB to form the pouch is not recommended [33]. Steffen et al has also reported use of an adjustable gastric band with a distal gastric bypass and the stomach in their technique was divided horizontally and very low leaving a huge gastric pouch [34]. Our technique however is completely different to these variations as described above.

With the combined procedure, a sequential action mechanism for EWL is to be expected. The EWL with RYGB will be effective at the beginning reaching a plateau after 12 - 18 months. The filling of the band at this time will result in further adjustable gastric pouch restriction thereby causing further weight loss. Also, the adjustable band will limit the volume of food intake, especially when restriction fades with time and weight regain would occur. The procedure thus combines the potential benefits of RYGBP and an AGB.

We already described our technique of the laparoscopic adjustable banded sleeve gastrectomy in one patient without any device-related perioperative complications [35]. Especially in the RYGB procedure, one may be concerned about possible band or port infection since, in contrast to the sleeve gastrectomy, both the gastric pouch and the small bowel are opened during the operation. Apart from the cefazoline given at induction, we did not take any special measures to avoid band contamination. One should of course try to limit excessive spillage of gastric or small bowel content during the operation by carefully opening the pouch or the bowel assisted by appropriate suction.

The AGB, however, has been associated with late complications, including slippage and erosion of the band. Since the band is placed through a small opening between the blood vessels immediately adjacent to the stomach and the lesser curve and fixed laterally with the gastric remnant both above and below the band, the chance of slippage is expected to be low. Whether or not late complications will occur remains to be seen.

## Conclusion

The insertion of an AGB is feasible and seems to be safe during a LRYGB at the *primary *operation, with no immediate major complications. We assume that combining an adjustable band to Roux-en-Y gastric bypass will lead to better excess weight loss results than those of a gastric bypass alone. It is hoped that this combined procedure will be most useful in the super-super obese (Body mass index > 60 kg/m^2^) patients. More patients with a long-term follow-up are necessary to provide definitive conclusions regarding long-term benefits and complications of this combined bariatric procedure.

## Competing interests

The authors declare that they have no competing interests.

## Authors' contributions

Study conception and design: *BD; *Drafting of manuscript: *BD, SVC, SA*; Acquisition of data: *EVD, SVC; *Analysis and interpretation of data: *EVD, SVC; *Critical revision: *BD, SVC, JPM*.

All authors read and approved the final manuscript.

## Pre-publication history

The pre-publication history for this paper can be accessed here:

http://www.biomedcentral.com/1471-2482/10/33/prepub
